# Performance of initial LI-RADS 2018 treatment response in predicting survival of patients with hepatocellular carcinoma following TACE: a retrospective, single-center cohort study

**DOI:** 10.1007/s00432-021-03603-9

**Published:** 2021-03-28

**Authors:** Krzysztof Bartnik, Joanna Podgórska, Grzegorz Rosiak, Krzysztof Korzeniowski, Jakub Giziński, Michał Sajdek, Tadeusz Wróblewski, Krzysztof Zieniewicz, Paweł Nyckowski, Olgierd Rowiński

**Affiliations:** 1 Doctoral School, Medical University of Warsaw, Warsaw, Poland; 2 Second Department of Radiology, Medical University of Warsaw, Ul. Banacha 1a, 02-097 Warsaw, Poland; 3 Department of General, Transplant and Liver Surgery, Medical University of Warsaw, Warsaw, Poland; 4 Department of General, Gastroenterological and Oncological Surgery, Medical University of Warsaw, Warsaw, Poland

**Keywords:** LI-RADS, Treatment response, TACE, HCC, Outcomes, Survival, Progression

## Abstract

**Purpose:**

Treatment response following transarterial chemoembolization (TACE) is frequently evaluated with Liver Imaging Reporting and Data System Treatment Response (LR-TR) algorithm, but its association with patients’ outcomes is not supported in the literature. The purpose of this study was to provide such data.

**Methods:**

A retrospective analysis of 99 TACE patients with stage A/B hepatocellular carcinoma according to Barcelona-Clinic Liver Cancer staging system was performed. Two radiologists assessed LR-TR, while a third radiologist re-assessed divergent results. Overall survival (OS) and time to disease progression (TTP) were the primary endpoints of the study, while the Cox proportional hazard model was used for outcome analyses.

**Results:**

Interobserver agreement was substantial between the two readers with *κ* = 0.69 (95% CI 0.58–0.81). The median OS in viable, equivocal, and non-viable groups were 27, 27, and 73 months, respectively (*p* < 0.001). However, after adjustment for confounding factors, there was no significant association between initial viable response and OS (HR 0.98 [95% CI 0.37–2.63], *p* = 0.97), while equivocal response remained statistically significant (HR 3.52. [95% CI 1.27–9.71], *p* = 0.015). No significant association was noted when viable and equivocal groups were analyzed in aggregate (HR 1.03 [95% CI 0.4–2.4], *p* = 0.96). The median TTP did not differ between non-viable and viable groups (23 vs 18 months, respectively; *p* = 0.98). None of the analyzed predictors was associated with TTP.

**Conclusion:**

Initial LR-TR response was not an independent predictor for OS nor TTP. The preliminary results suggest the necessity for more aggressive management of equivocal patients.

## Introduction

Hepatocellular carcinoma (HCC) represents an emerging challenge for health care providers given its increasing incidence and high mortality (Dimitroulis et al. 2017). Among various treatment modalities, transarterial chemoembolization (TACE) is frequently used as the initial treatment in patients with unresectable HCC (Llovet et al. 2002; Marrero et al. 2018). To correctly evaluate treatment response, experienced health care providers are needed, which almost always need to be supported by a series of imaging examinations and laboratory tests (Mehta 2020). To standardize interpretation, several treatment response algorithms are available including the WHO and mRECIST criteria (Bruix et al. 2001; Forner et al. 2009; Lencioni and Llovet 2010). Given the increasing use of TACE and ablations, great investments have been made in the development of robust, more effective algorithms to evaluate response to locoregional therapies. This has led to the development of the HCC-specific treatment response algorithm (LR-TR), as an expansion of the Liver Imaging Reporting and Data System (LI-RADS), aimed specifically at assessing response after HCC locoregional therapy (Chaudhry et al. 2020a). The LI-RADS is currently the most popular system of lesion classification in CT and MRI studies in patients with increased risk of HCC (Chernyak et al. 2018; Kim et al. 2019; Aslam et al. 2020a; Rosiak et al. 2018b). There are many studies showing the high specificity and sensitivity of LI-RADS for HCC reporting (Rosiak et al. 2018a; Furlan 2019).

The LR-TR was primarily designed to increase inter-reader agreement in reporting and improve decision-making processes (Voizard et al. 2019a). It is a relatively new algorithm that offers new opportunities to further improve HCC patient care, but still requires in-depth research (Aslam et al. 2020b; Do and Mendiratta-Lala 2020). Previous studies have shown that tumor response after TACE (assessed using the mRECIST or WHO criteria) is associated with patient outcomes/overall survival (Shim et al. 2012). Considering that the LR-TR has not yet been shown to be a useful predictor of patient outcomes following TACE, other systems are currently more frequently used to assess treatment response in the clinical trial setting (Nishino et al. 2010; Meng et al. 2018; Vogel et al. 2018).

The purpose of this study is to assess the initial LR-TR algorithm response as a potential predictor of outcomes in patients who are not candidates for resection or transplantation and who are undergoing repeated TACE sessions as HCC therapy. Overall survival and time to disease progression were analyzed as the main outcome variables.

## Materials and methods

In this retrospective, single-centre study we analyzed the data of patients who underwent TACE as first line therapy. The first primary endpoint was overall survival; the second primary endpoint was time to progression in patients who achieved a favorable treatment response. Approval for the study was obtained from the local Institutional Ethical Committee of Human Experimentation and the study was performed in accordance with the current version of the Declaration of Helsinki.

### Study population

**Fig. 1:**
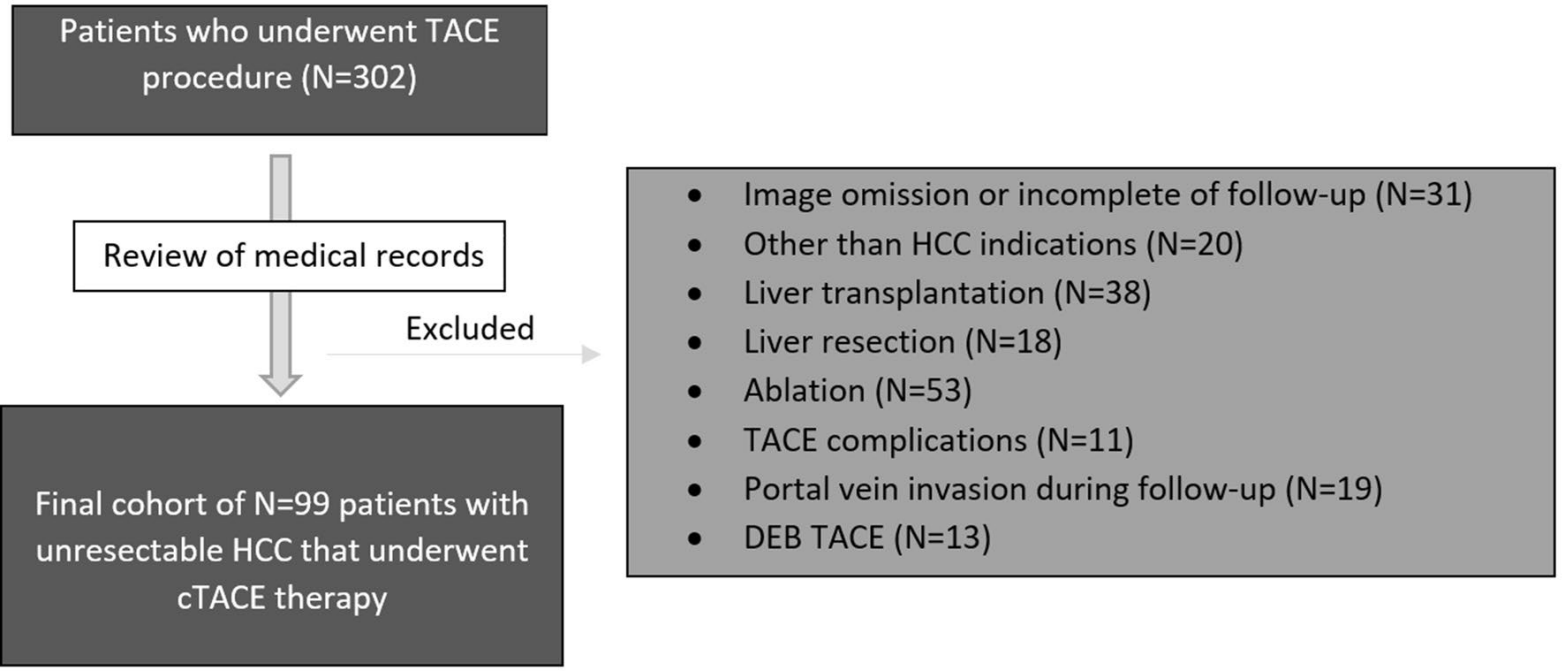
Flowchart of study population enrollment

A search of the internal database was performed for patients undergoing TACE between March 3, 2016 and January 26, 2018. Three radiologists reviewed the records and imaging via the picture archiving and communication system (PACS). Figure 1 shows a summary of the inclusion and exclusion criteria and the final study population.

The inclusion criteria were as follows: (1) patients had at least one index HCC lesion confirmed by imaging or pathology; (2) patients were undergoing TACE as initial HCC therapy; (3) patients had good liver function (i.e., Child–Pugh A or B), without the presence of vascular tumor thrombus or metastatic disease; and (4) patients had an available dynamic contrast enhanced CT or MRI liver examination within 90 days after the first TACE cycle.

A total of 203 patients were excluded based on the following criteria: (1) other (non-TACE) HCC-specific treatment including ablation or resection before TACE or during follow-up period; (2) liver transplantation before procedure or during follow-up period; (3) history of malignant neoplasm other than HCC; (4) uncontrolled functional or metabolic disease before the procedure or during the follow-up period (including portal vein thrombosis); (5) TACE complications (e.g., significant post-procedural liver decompensation); and (6) incomplete clinical and follow-up data.

All patients underwent a series of laboratory examinations before the initial TACE session, including a liver function panel. The following variables were analyzed: age, sex, underlying etiology of chronic liver disease, serum total bilirubin, albumin, creatinine, international normalized ratio (INR), α-fetoprotein level, Barcelona-Clinic Liver Cancer (BCLC) stage, and Child–Pugh–Turcotte (CPT) score. The diagnosis of HCC was made using imaging criteria (LI-RADS).

### TACE technique

All patients underwent standard conventional TACE procedures. After obtaining femoral access, angiographic runs were performed to confirm hepatic and tumoral blood supply. Vessels feeding target lesions were selectively catheterized with a microcatheter and 20–40 mL of a mixture of lipiodol and doxorubicin in a 1:1 ratio was slowly injected until arterial flow stasis was observed.

TACE was repeated after 4–6 weeks when indicated and feasible. A standard embolization cycle consisted of two (or three, if indicated) TACE sessions and subsequent CT/MRI work-up. One patient had only one TACE procedure before the first treatment response (LR-TR) assessment.

### Postprocedural work-up and follow-up

All patients underwent a multiphasic CT (or optional MRI study) between 30 and 90 days after completion of the TACE cycle as recommended by the LR-TR guidelines (Chernyak et al. 2018). Image sections were acquired using a multi-slice CT scanner with non-ionic contrast medium during the precontrast phase, the late arterial phase, the portal venous phase, and the equilibrium phase. All imaging examinations were compliant with LI-RADS 2018 technical recommendations. If a favorable treatment response with no viable tumor tissue was visualized after the TACE cycle, patients were followed up by serial imaging and measurements of serum α-fetoprotein concentration until HCC recurrence. If a viable tumor was present during follow-up, a multidisciplinary team meeting was held for consensus regarding management and further treatment options.

### Imaging analysis

Two independent reviewers (with 5 years’ and 8 years’ experience in liver imaging, respectively) evaluated post-treatment images. Both observers were informed that study patients had undergone TACE for HCC but were blinded to the clinical, laboratory, and survival data. All imaging features were analyzed according to the LR-TR (LI-RADS v. 2018) and recorded by the two radiologists. Another radiologist with 11 years’ experience of liver imaging re-evaluated any discordant results and drew the final conclusion.

All patients were divided into three subgroups according to the LR-TR, with response defined as follows:

1. LR-TR non-viable—no arterial phase hyperenhancement (APHE) and/or the presence of expected treatment-specific enhancement pattern.
2. LR-TR equivocal—atypical enhancement pattern, not meeting criteria for non-viable or viable category.
3. LR-TR viable—APHE or washout appearance or enhancement similar to pretreatment.

If a patient had more than one observation, each representing different responses, the final response category was reported in aggregate by choosing the one reflecting the less favorable response. In patients who did not achieve an initial non-viable response, the subsequent overall treatment response was defined using pre-existing radiology reports, with lack of tumoral enhancement as a criterion of favorable treatment response.

The date of the first TACE procedure was adopted as an index day. The end of the follow-up period was defined as the time of death or last clinical follow-up (September 24, 2020). In patients who achieved a favorable treatment response, time to progression was defined as the interval between achieving non-viable tumor status and the date of reported progression.

### Statistical analysis and artwork

Statistical analyses were performed using SAS software (Statistical Analysis System version 9.4, SAS Institute Inc., Cary, NC, USA). Categorical variables are shown as counts and percentages. The kappa coefficient (κ) was used to assess the interobserver agreement for LR-TR responses. Fisher’s exact and Kruskal–Wallis tests were used to compare differences between study subgroups. In addition, we used the Kaplan–Meier method together with the log-rank and Peto–Peto–Prentice tests with Šidák correction to compare differences between survival curves. A Cox proportional hazard model was used for univariate and multivariate survival analyses.

## Results

### Demographics

Patients’ baseline characteristics are shown in Table 1. The final study cohort consisted of 99 patients (27 women and 72 men); the median patient age was 66 years (IQR 59–73; range 25–88). The majority of patients had preserved liver function (CPT A, 87%), and 48 (48%) had intermediate stage (B) HCC according to the BCLC staging. In total, 28 (28%) patients had baseline α-fetoprotein levels greater than 200 ng/mL. After disqualification from further locoregional treatment, 18 (18%) individuals were subsequently treated with sorafenib.

**Table 1:** Demographic and clinical characteristics of patients

Baseline characteristic	No. of patients	(%) Or median, (range)
Age, years		
Median	67 (59–73)	(25–88)
< 60	31	30.10
> 60	67	68.37
Gender		
Male	72	72.73
Female	27	27.27
Chronic liver disease etiology		
Viral	55	53
Alcoholic	26	25
Mixed	4	4
Other	18	18
CPT class		
A	87	87.88
B	12	12.12
BCLC stage		
A	51	51.52
B	48	48.48
Serum AFP		
< 200 ng/mL	71	71.72
≥ 200 ng/mL	28	28.28
Albumin, g/L		4.1 (2.5–5.2)
Creatinine, mg/dL		0.87 (0.54–1.56)
Total bilirubin, umol/L		0.95 (0.24–5.6)
INR		1.15 (0.91–2.45)
ALT, IU/L		49 (12–348)
AST, IU/L		57.0 (20–408)
Number of TACE procedures		3 (1–11)
1	1	01.01
2	40	40.40
3	21	21.21
> 3	37	37.37
Sorafenib		
No	81	81.82
Yes	18	18.18

*CPT* Child–Pugh–Turcotte, *BCLC* Barcelona-Clinic Liver Cancer, *INR* international normalized ratio, *AFP* α-fetoprotein, *ALT* alanine aminotransferase, *AST* aspartate aminotransaminase

### TACE outcomes and LR-TR responses

A median of two TACE sessions (1–3) were performed before assessment of the first cycle response. Initial post-embolization imaging examinations were performed using CT in 94 (94%) patients, whereas 6 (6%) patients underwent MRI. Inter-reader agreement showed substantial correlation between the two readers using LR-TR (*κ* = 0.69; 95% CI 0.58–0.81). Table 2 shows the interobserver agreement between the two readers and the final conclusion after re-evaluation of divergent results by the third radiologist. Six patients could not be definitively characterized and fell into the LR-TR equivocal category. Table 3 shows pretreatment patient characteristics in subgroups defined by consensus LR-TR response. These subgroups differed significantly in terms of BCLC stage, baseline albumin concentration, aspartate aminotransferase (AST) level and proportion of patients included in the sorafenib program.

**Table 2:** Interobserver agreement between the two readers and the final conclusion after re-evaluation of divergent results by the third radiologist using LR-TR (LI-RADS v. 2018)

Reader 2	Reader 1			
	Nonviable	Equivocal	Viable	Total
Nonviable	34 (34.4%)	4 (4.04%)	1 (1.01%)	39 (39.39%)
Equivocal	6 (6.06%)	3 (3.03%)	5 (5.05%)	14 (14.4%)
Viable	0 (0%)	2 (2.02%)	44 (44.44%)	46 (46.46%)
Total	40 (40.40%)	9 (9.09%)	50 (50.51%)	99 (100%)

Final LR-TR category	No. patients
Nonviable	39 (39.39%)
Equivocal	6 (6.06%)
Viable	54 (54.55%)
Total	99 (100%)

**Table 3:** Pretreatment patient characteristics in subgroups defined by LR-TR algorithm

Baseline characteristic	Nonviable	Equivocal	Viable	*p*
	39 (39.39%)	6 (6.06%)	54 (54.55%)	
Age, years				
< 60	14 (35.9%)	3 (50%)	15 (27.78%)	0.4115
> 60	25 (64.1%)	3 (50%)	39 (72.22%)	
Gender				
Male	28 (71.79%)	5 (83.33%)	39 (72.22%)	0.0622
Female	11 (28.21%)	1 (16.67%)	15 (27.78%)	
CPT class				0.16
A	37 (94.87%)	5 (83.33%)	45 (83.33%)	
B	2 (5.13%)	1 (16.67%)	19 (16.67%)	
BCLC stage				
A	32 (82.05%)	3 (50%)	16 (29.63%)	** < 0.0001**
B	7 (17.95%)	3 (50%)	38 (70.37%)	
Serum AFP				
< 200 ng/mL	31 (79.49%)	4 (66.67%)	36 (66.67%)	**0.0249**
≥ 200 ng/mL	8 (20.51%)	2 (33.33%)	18 (33.33%)	
Albumin, g/L	4.3 (3–5.2)	3.78 (2.5–4.9)	3.95 (2.7- 5.0)	**0.0295**
Creatinine, mg/dL	0.91 (0.54–1.38)	0.77 (0.65–1.16)	0.74 (0.56–1.56)	0.4046
Total bilirubin, umol/L	0.76 (0.24–4.04)	1.46 (0.51–2.18)	1.03 (0.56–1.56)	0.1689
INR	1.16 (0.92–2.45)	1.15 (1.04–1.29)	1.15 (0.91–1.66)	0.9015
ALT, IU/L	49 (19–303)	56 (20–85)	53.5 (12–348)	0.8272
AST, IU/L	46 (20–243)	54 (32–129)	64 (21–408)	0.0356
Number of TACE procedures				
1	0 (0%)	0 (0%)	1 (1.85%)	0.3093
2	22 (56.41%)	2 (33.33%)	16 (29.63%)	
3	8 (20.51%)	1 (16.67%)	12 (22.22%)	
> 3	9 (23.08%)	3 (50%)	25 (46.3%)	
Sorafenib				
No	38 (97.44%)	5 (83.33%)	38 (70.37%)	**0.0002**
Yes	1 (2.56%)	1 (16.67%)	16 (29.63%)	

Statistically significant differences are indicated in bold (p < 0.05)

*CPT* Child–Pugh–Turcotte, *BCLC* Barcelona-Clinic Liver Cancer, *INR* international normalized ratio, *AFP* α-fetoprotein, *ALT* alanine aminotransferase, *AST* aspartate aminotransaminase

At the time of first check-up and re-evaluation by the multidisciplinary team, 58 (59%) individuals had not achieved the desired treatment response. Embolization sessions were sufficient to achieve a favorable response in 18 (31%) of these patients. Ultimately, repeated TACE sessions were sufficient to achieve a favorable treatment response in a total of 59 (60%) of the 99 patients.

## Comparison of endpoints according to LR-TR responses

### Survival analysis

The median follow-up duration was 29 months (IQR 17–38; 4–81). At the time of analysis 59 (60%) of the patients had died and 40 (40%) patients were censored (with vital status ascertained at the time of last follow-up).

**Fig. 2:**
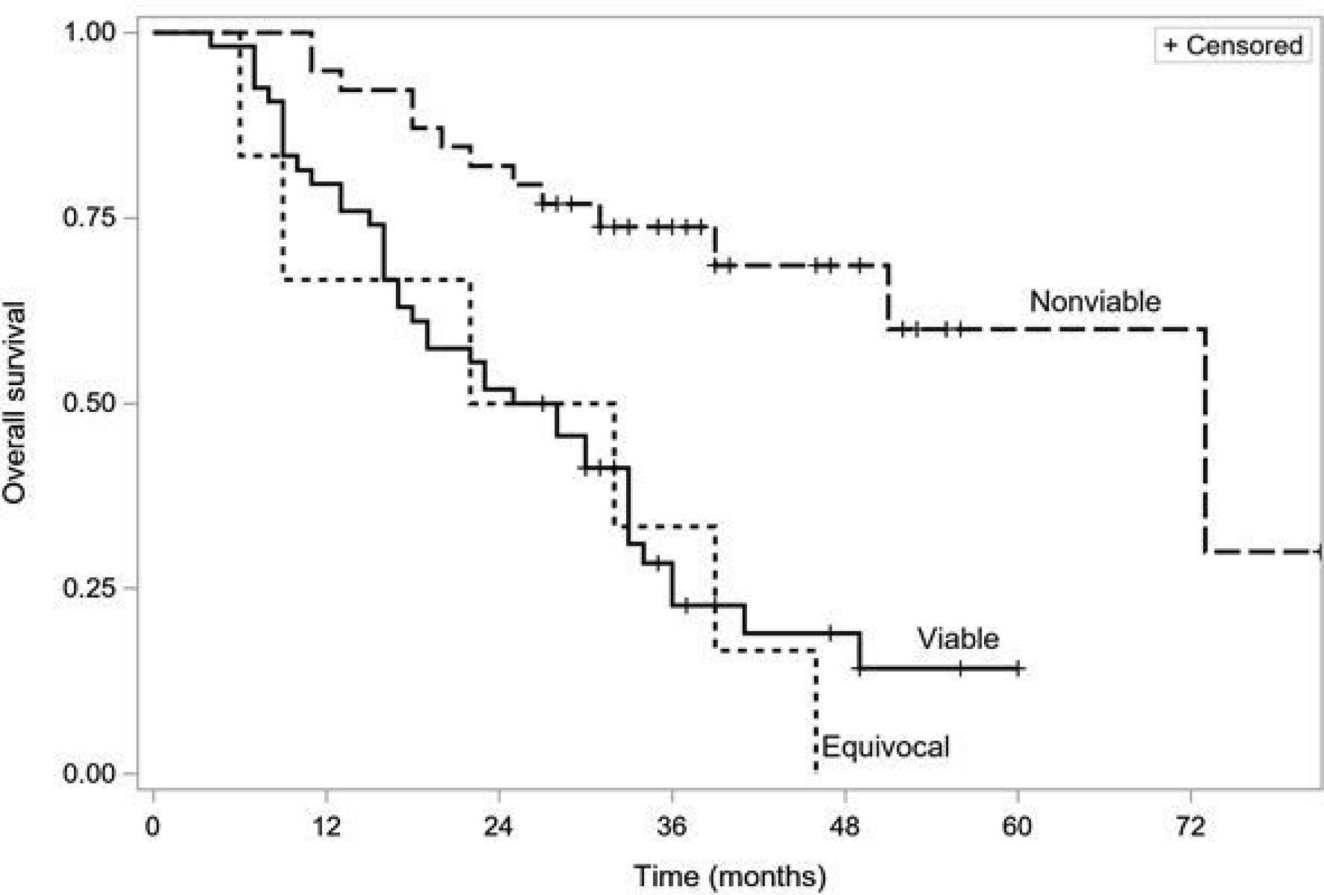
Kaplan–Meier survival curves of patients using the LR-TR criteria, non-viable vs equivocal *p* = 0.0001, non-viable vs viable—*p* = 0.0002, equivocal vs viable—*p* = 0.045 (Peto–Peto–Prentice test, Šidák correction)

Figure 2 shows the Kaplan–Meier survival curves of patients using the LR-TR criteria. The median survival times in viable, equivocal, and non-viable groups were 27, 27, and 73 months, respectively (*p* < 0.001). The Peto–Peto–Prentice test with Šidák correction for multiple comparisons showed a significant difference in overall survival between the LR-TR non-viable, and viable groups (*p* < 0.001) and between the non-viable and equivocal groups (*p* < 0.001). Overall survival differed between the viable and equivocal groups (*p* = 0.045), but there was a significant overlap in the confidence intervals for the Kaplan–Meier estimates.

### Time-to-progression analysis

**Fig. 3:**
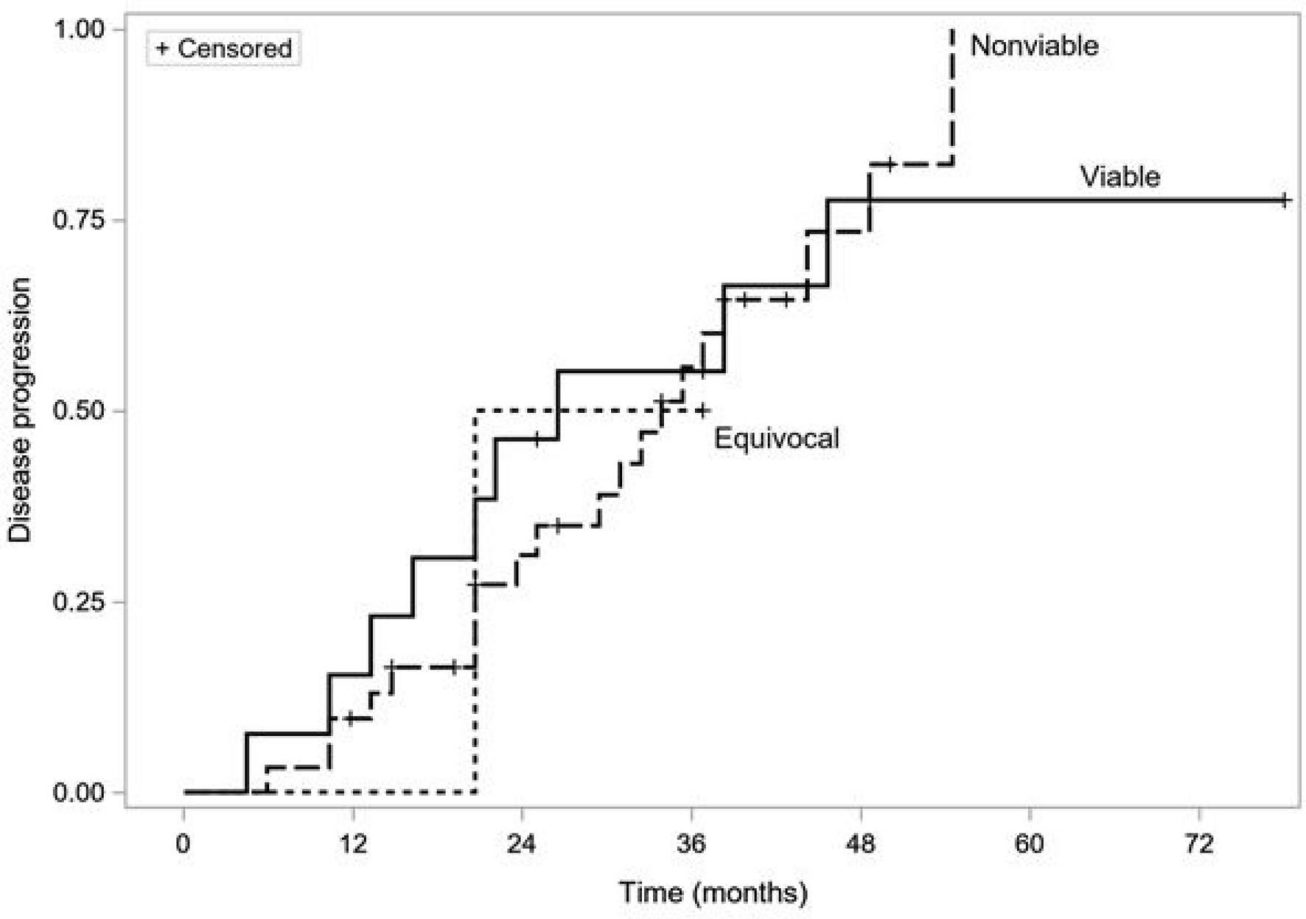
Kaplan–Meier time-to-progression curves of patients using the LR-TR criteria, *p* = 0.98 (log-rank test)

Among a subgroup of 59 patients who achieved favorable responses, data regarding time to progression were recorded in 46 (78%) individuals during the median 18 months of follow-up. In total, 30 patients had confirmed progression and 16 were censored. There was no significant difference in time to progression with respect to LR-TR response after the first TACE cycle (23 vs 18 months in the non-viable and viable response groups, respectively; *p* = 0.98). Figure 3 shows time-to-progression curves of patients using the LR-TR response criteria.

### Univariate outcome prediction on the basis of selected prognostic factors

Table 4 shows hazard ratios (HRs) for the prediction of overall survival for prognostic factors generated from the univariate Cox regression model. BCLC stage, number of target lesions, initial LR-TR treatment response, overall favorable response, albumin concentration, and treatment with sorafenib were found to be statistically significant prognostic variables. None of the analyzed factors proved to be a significant explanatory variable for time to progression in the univariate analysis (data not shown).

**Table 4:** Hazard ratios for the prediction of overall survival for prognostic factors generated from the univariate analysis

Parameter	Hazard ratio	95% confidence interval	*p* value
Age	1.02	0.99–1.05	0.16
Female	1.06	0.59–1.91	0.85
BCLC stage A	1.00		Reference
BCLC stage B	2.64	1.55–4.50	** < 0.0001**
Nonviable	1.00		Reference
Equivocal	4.77	1.78–12.78	**0.002**
Viable	3.76	1.96–7.23	** < 0.0001**
Favorable overall response	0.21	0.12–0.36	** < 0.0001**
CPS class A	1.00		Reference
CPS class B	1.84	0.93–3.65	0.08
Albumin	0.58	0.36–0.94	**0.027**
Serum AFP	1.33	0.76–2.36	0.32
AST	1.00	1.00–1.01	0.22
Sorafenib	2.46	1.37–4.41	**0.003**

Statistically significant differences are indicated in bold (p < 0.05)

*BCLC* Barcelona-Clinic Liver Cancer, *CPT* Child–Pugh–Turcotte, *AFP* α-fetoprotein, *AST* aspartate transaminase

### Survival prediction on the basis of LR-TR response, adjusted by covariates

The multivariate Cox regression model exploring prognostic factors for overall survival is shown in Table 5. The HRs were adjusted for significant predictors (*p* < 0.05) derived from univariable Cox regression analyses (Table 4). Despite the initial LR-TR category being a significant predictor of overall survival on univariate analysis (HRs 4.77 and 3.76 for the equivocal and viable groups, respectively), adjusted analysis showed no significant differences between initial non-viable and viable response groups in terms of overall survival (adjusted HR 0.98, 95% CI 0.37–2.63; *p* = 0.97). After adjustment, multivariate analysis revealed a significant independence of the best overall treatment response, albumin concentration and LR-TR equivocal response as predictors of overall survival. This multivariate model for prediction of overall survival resulted in a c-statistics value of 0.85 (95% CI 0.76–0.91). The results did not differ significantly when LR-TR viable and equivocal responses were analyzed in aggregate (HR 1.03 [95% CI 0.4–2.4], *p* = 0.96).

**Table 5:** Hazard ratios for the prediction of overall survival for prognostic factors generated from the multivariate analysis

Parameter	Hazard ratio	95% confidence interval	*p* value
BCLC A	1.00		Reference
BCLC B	1.70	0.91–3.17	0.094
Nonviable	1.00		Reference
Equivocal	3.52	1.27–9.71	**0.0152**
Viable	0.85	0.31–2.32	0.7482
Favorable overall response	0.22	0.09–0.51	**0.0005**
Albumin	0.59	0.36–0.97	**0.0387**
Sorafenib	1.35	0.72–2.53	0.3428

Statistically significant differences are indicated in bold (p < 0.05)

*BCLC* Barcelona-Clinic Liver Cancer

## Discussion

In the present study, an initial LR-TR response was assessed as a potential predictor of survival in HCC patients treated with TACE. The results indicate that the worse outcomes observed in HCC patients who did not achieve a non-viable response after the initial treatment cycle are due to unfavorable baseline characteristics rather than the prognostic impact of the initial treatment response itself. It should be noted that the analyzed groups differ significantly in terms of baseline hepatic function (namely, albumin concentration) and BCLC stage, although these variables were treated as possible confounders in a multivariate analysis. An analysis that adjusted for confounding factors showed that initial viable response did not improve the capability of combining tumor staging (BCLC), liver function (albumin) and best overall radiological response to predict overall survival. Notably, multivariable analysis pointed towards a negative effect of equivocal response in terms of overall survival; however, the number of patients was low (*n* = 6). We decided to re-evaluate the model and to analyze equivocal and viable groups in aggregate, but noted no significant difference. Nevertheless, our data demonstrated non-inferiority in overall survival for initial viable vs equivocal groups indicating the clinical need for careful planning of further management of equivocal subjects. Moreover, the results of the present study confirm that a combination of HCC stage and underlying liver function together with treatment response criteria allows for efficient identification of HCC patients with poor outcomes, in line with numerous previous studies (Takayasu et al. 2006; Shim et al. 2012; Pinato et al. 2016; Han et al. 2020).

Notably, the baseline characteristics of patients who did not achieve a viable response were not reported in previous studies assessing outcomes in patients classified by LR-TR. A recent study by Zhang et al. analyzed the association between LR-TR response and overall survival in patients undergoing radiofrequency ablation (RFA) (Zhang et al. 2020). The study, in which the vast majority of patients underwent a single ablation session, showed that LR-TR response was associated with overall survival, while patients with an LR-TR viable response had significantly lower overall survival than other patients. However, the authors did not provide data regarding baseline prognostic factors for each analyzed group separately, which could significantly affect the study endpoints. Notably, that study showed no difference in overall survival between the non-viable and equivocal groups. By contrast, the median overall survival did not differ between the viable and equivocal groups in our study. Our findings may be partially explained by previous studies, which reported a high prevalence of viable tumor tissue at histopathology when the treated tumor was assigned to the LR-TR equivocal category. Choudhry et al. showed that five of six lesions that were classified as LR-TR equivocal were incompletely necrotic at histopathology (Chaudhry et al. 2020). Elsewhere, Shropshire et al. reported that the 71% of lesions characterized as LR-TR equivocal in their study were incompletely necrotic at pathological examination, and that these lesions may, therefore, warrant additional treatment (Gervais 2019). It must be stated that, despite the results of the current study contradicting the prognostic relevance of initial treatment response as a predictor of poor outcomes in patients undergoing TACE, they do not undermine the role of overall radiological assessment as an independent predictor of overall survival following repeated embolization sessions. This finding was expected as a substantial number of patients with initial viable status achieved a favorable response in subsequent TACE sessions.

Because the current study aimed to investigate the prognostic effect of initial LR-TR response on survival outcomes, and due to the small sample size, the study is underpowered to draw conclusions regarding time to progression for each group. However, a subgroup analysis showed that TTP intervals did not differ between patients with initial viable and non-viable LR-TR response, nor were they associated with any of the potential risk factors analyzed. Given the low number of patients, these data should be considered preliminary and studies with larger study groups (possibly multicenter) will be needed to assess those differences. Nevertheless, we showed time-to-progression intervals in patients with different initial LR-TR responses following TACE—to our knowledge, this has not previously been reported in the literature.

Considering the unfavorable prognoses of patients with viable tumor tissue the goal is to treat HCC so that no viable tumor tissue is present at the follow-up imaging examination. The LI-RADS algorithm assumes that radiological findings are an imperfect measure of complete tumor necrosis (Chernyak et al. 2018; Voizard et al. 2019b). It is still unclear how responses measured by LR-TR criteria reflect patient outcomes, given the risk of minimal residual disease and tumor recurrence (Piñero et al. 2020). A few pilot studies that aimed to investigate the performance of the LR-TR for prediction of tumor necrosis following locoregional treatment have been published; however, the majority investigated only a single session of locoregional therapy (namely, transcatheter bland embolization or ablation), and a low number of patients received multiple treatment sessions (Gervais 2019; Chaudhry et al. 2020). Despite this focus on the homogeneity of the study cohorts, studies have failed to show the impact of repeated treatment sessions on tumor viability.

The main goal of all HCC treatment response algorithms is to support clinical decision making by improving inter-reader agreement and evaluating the imaging criteria of viable tumor tissue, mainly via assessing the presence of enhancing tumor tissue (Abdel Razek et al. 2020). In general, the inter-reader agreement in our study was substantial using the LR-TR algorithm, in line with previous studies (Chaudhry et al. 2020; Zhang et al. 2020). The algorithm allows for reproducible identification of patients who are likely to benefit from subsequent treatment sessions. However, LR-TR is not a true surrogate for quantifying patients’ outcomes and there are clearly other factors involved in survival prognosis, including pretreatment tumor stage, underlying liver disease and deterioration of liver function following treatment. Future outcome analysis would benefit from combining the LR-TR with baseline and post-treatment clinical data as well as existing prognostic scoring systems for patients receiving TACE for hepatocellular cancer, such as the hepatoma arterial-embolization prognostic (HAP) score or assessment for retreatment with TACE (ART) score (Kadalayil et al. 2013; Hucke et al. 2014).

### Limitations

The present study analyzed a single-center cohort using a retrospective study design, which could potentially contribute to selection bias. The standard TACE cycle consisted of two or three sessions of TACE, however, one patient was treated with only one TACE session before evaluation of treatment response. It is worth noting that, although 39% of patients achieved non-viable treatment response at the time of first treatment assessment, many patients needed subsequent embolization sessions. Such heterogeneity reflects real-life scenarios, where different HCC lesions show different susceptibility to treatment. This potentially results in selection bias from initially advanced disease. Notably, several previous studies have shown conflicting results, with some finding that the initial response cannot predict long-term survival, while others report the opposite (Gillmore et al. 2011; Georgiades et al. 2012; Liu et al. 2014; Kim et al. 2015). It has been suggested that those discrepancies can be at least partially explained by the aforementioned bias resulting from patient selection criteria. Of note, we found that a non-viable treatment response seems to be more difficult to achieve for larger tumors and for patients with multiple target lesions. We conclude that, in patients with more advanced disease, the best overall response correlates better with treatment outcomes, as there is weak concordance between initial and overall best response, in line with the findings of Wenjun Wang et al. (2015). This finding is consistent with those of previous studies that analyzed the association between LT-TR responses following TACE and degree of tumor necrosis at histopathology. Future studies would benefit from showing an association between baseline patient characteristics and the number of TACE procedures needed to achieve a favorable radiological tumor response measured by the LR-TR.

It must be stated that, in patients who did not achieve an initial non-viable status, subsequent imaging studies were not re-evaluated and the overall treatment response was simply specified using pre-existing reports. This approach assumed the lack of APHE as a sole indicator of a favorable response, rather than the LR-TR status. We acknowledge that such an approach can induce inter-reader agreement bias; however, previous data emphasized that APHE is the feature most consistently associated with residual tumor viability and that it shows the greatest inter-reader agreement among all post-embolization features in different treatment response algorithms (Shim et al. 2012; Tang et al. 2018; Gervais 2019; Abdel Razek et al. 2020).

Finally, there was substantial homogeneity of imaging modalities in the study cohort; however, not only CT but also MRI (in 6% of patients) was used to assess post-treatment response. Of note, in LI-RADS, assessment of the response to treatment in CT and MRI are treated equally; however, in conventional TACE, the imaging modality may significantly affect the quality of the assessment. In CT imaging, the intratumoral lipiodol deposition, which facilitates localization of the embolic material within the tumor, may obscure the enhancement caused by residual viable tumor tissue (Chen et al. 2016; Dioguardi Burgio et al. 2019). This problem does not appear on MRI, where the low T1 signal of the material does not mask the enhancement (De Santis et al. 1997). It would be interesting to compare the outcomes of TACE procedures in patients assessed with CT versus MRI, possibly adding ancillary imaging features (Kim et al. 2020). Additionally, although the majority of imaging examinations were performed with in-house scanners, substantial numbers were not (data not shown). We acknowledge that different CT and MRI scanners could potentially influence the interpretation of imaging features and thus affect the interobserver agreement. However, this scenario represents real-life clinical practice, in which each patient, for life reasons, may undergo check-up examinations in outpatient settings outside tertiary health centers, using many different scanners at the place of residence. Such a strategy is employed in our center to improve patients’ quality of life, as many patients live in distant locations and it would be highly inconvenient to perform repeated check-ups in a centralized facility.

## Conclusion

Increasing understanding of treatment outcomes in different LR-TR groups will help to develop more specific approaches for each particular subgroup of patients. First, our observations suggest that the initial LR-TR response category is not an independent predictor of overall survival in HCC patients treated with TACE, and poor outcomes for HCC patients who did not achieve a non-viable response after the initial treatment cycle may be due to unfavorable baseline characteristics. We conclude that, in HCC patients treated with TACE, the initial LR-TR response category is inferior to the best overall treatment response in predicting overall survival. Additionally, our findings also suggest the necessity for more aggressive management of equivocal patients, however, more research is needed in this regard. Perhaps these two groups can be somehow combined in the future to simplify the classification.

## Data Availability

The relevent data and materials can be requested from the authors.
